# Microvascular Changes After Conbercept Intravitreal Injection of PDR With or Without Center-Involved Diabetic Macular Edema Analyzed by OCTA

**DOI:** 10.3389/fmed.2022.797087

**Published:** 2022-03-22

**Authors:** Wei Lin, Meng Feng, Tingting Liu, Qingxu Wang, Wenqi Wang, Xiao Xie, Wenhao Li, Jitian Guan, Zhongyu Ma, Tong Liu, Qingjun Zhou

**Affiliations:** ^1^School of Basic Medicine, Shandong First Medical University and Shandong Academy of Medical Science, Shandong Provincial Hospital Affiliated to Shandong First Medical University, Jinan, China; ^2^Eye Hospital of Shandong First Medical University (Shandong Eye Hospital), Jinan, China; ^3^State Key Laboratory Cultivation Base, Shandong Provincial Key Laboratory of Ophthalmology, Qingdao, China; ^4^Shandong Eye Institute, Shandong First Medical University and Shandong Academy of Medical Sciences, Qingdao, China; ^5^School of Ophthalmology, Shandong First Medical University, Jinan, China; ^6^Jinan Mingshui Eye Hospital, Jinan, China; ^7^The First Clinical Medical College of Shandong University of Traditional Chinese Medicine, Jinan, China; ^8^Computer Department of Southwest University of Science and Technology, Mianyang, China; ^9^Department of Medicine, Xizang Minzu University, Xianyang, China; ^10^Qingdao Eye Hospital of Shandong First Medical University, Qingdao, China

**Keywords:** proliferative diabetic retinopathy (PDR), conbercept, foveolar avascular zone (FAZ), central retinal thickness (CRT), best corrected visual acuity (BCVA), optical coherence tomography angiography (OCTA)

## Abstract

**Purpose:**

To investigate the intravitreal injection of conbercept as a treatment strategy for proliferative diabetic retinopathy (PDR) with or without center-involved diabetic macular edema (CI-DME) and evaluate its effect on the microvascular changes in the eyes.

**Methods:**

In this prospective study, 43 patients including 29 cases (56 eyes) in CI-DME with PDR patients, and 14 cases (26 eyes) in the non-center involving diabetic macular edema (NCI-DME) with PDR patients were involved in this study. The best corrected visual acuity (BCVA), central retinal thickness (CRT), foveolar avascular zone (FAZ), and macular capillary vessel density (VD) of the superficial retinal capillary plexus (SCP) and deep retinal capillary plexus (DCP) were assessed before and after conbercept treatments for 1, 3, or 6 months.

**Results:**

The BCVA was significantly increased after conbercept treatment in the eyes of CI-DME patients. After 6 months of treatment with the conbercept, microvascular density of the inferior area in SCP and the central fovea area in DCP increased significantly, regardless of the central fovea involvement. The effect of the conbercept treatment on the VD of NCI-DME was higher than that of CI-DME. Then, after 6 months of treatment, the CRT of patients with CI-DME and NCI-DME were decreased significantly.

**Conclusions:**

In this study, an intravitreal injection of conbercept significantly improved vision, alleviated macular edema in patients with DME. Conbercept treatment also altered the microvascular density in the retina.

## Introduction

Diabetic retinopathy (DR) is the common microvascular complications of diabetes. It is the main cause of visual impairment worldwide. Millions of patients with diabetes may progress to vision-threatening retinopathy, defined as proliferative diabetic retinopathy (PDR) or macular edema (1–4). PDR combined with different degrees of diabetic macular edema (DME) is one of the most common causes of visual impairment.

Intravitreal anti-vascular endothelial growth factor (VEGF) therapy is a first-line treatment for DME (5–11). Ranibizumab is an anti-VEGF inhibitor. Recently, the randomized Diabetic Retinopathy Clinical Research Network (DRCR.net) Protocol S clinical trial showed that ranibizumab intravitreal therapy was effective for treating PDR (12). Conbercept (KH902; Chengdu Kanghong Biotech Co., China) is a recombinant anti-VEGF protein. It has been produced by the expression system of Chinese hamster ovary (CHO) cells (13, 14). Studies proved the efficacy and safety of conbercept injections for the treatment of DME and PDR (15, 16).

It is important to evaluate the macular perfusion of repeated injections that are often required for long-term treatment. Some studies have analyzed macular capillary perfusion by fundus autofluorescence (FFA). But FFA requires manual measurement of macular capillary perfusion, and its analysis results are easily affected by factors, such as leakage, hemorrhage, and image quality (17–21). Optical coherence tomography angiography (OCTA) is a newly developed technology of fundus angiography, which is used to stratify the vessels of the retina and choroid, and has become an important instrument for the diagnosis and treatment of diabetic retinopathy (2, 22). Thus, OCTA can be used to observe the therapeutic effect of anti-VEGF drugs more precisely (10, 11, 15, 23). A few prior studies compared the effect of conbercept on DME by OCTA, and the results were controversial (22–24).

In this study, we aimed to investigate the intravitreal treatment of conbercept for PDR with or without center-involved DME (CI-DME) and evaluated its effect on the microvascular changes in retina.

## Methods

### Ethical Approval and Study Registration

Patients with type 2 diabetes were recruited from Eye Center, Shandong Eye Hospital in China, from November 2019 to November 2020. In total, 82 eyes of 43 patients were diagnosed as DME by FFA and optical coherence tomography (OCT). Study approval was obtained from by the Institutional Ethics Committee of Shandong eye hospital (approval No. 2019S001) and the study followed the Declaration of Helsinki. According to whether the central fovea involved patients were divided into two groups: center-involving DME (CI-DME) with PDR patients and non-center involving DME (NCI-DME) with PDR patients. All of them are naive patients who have never been diagnosed and treated. Informed consent was obtained from all patients who received intravitreal conbercept injection (5–7, 25, 26).

### Inclusion and Exclusion Criteria

The patients recruitment criteria were as follows: (1) BCVA needed to have a measure of 20/400 or better, and pre-treatment and post-treatment OCTA images needed to be of good quality (≥6/10 quality score). (2) Patients with type 2 diabetes aged ≥ 18 years. (3) Patients with PDR, retina neovascularization by FFA. Patients with DME, retinal thickening within two disc diameters of the central fovea and can be either focal or diffuse. Patients with CI-DME, retinal thickening in the macula that involves a central subfield zone is 1 mm in diameter (11, 22, 23). Patients with NCI-DME, retinal thickening in the macula that does not involve a central subfield zone is 1 mm in diameter (5–7). (4) The patients who met the criteria of anti-VEGF treatment guidelines would receive at least 3 months consecutive intravitreal conbercept. (5) No history of ocular trauma and surgery and no history of fundus related diseases or treatment.

Exclusion criteria were as follows: (1) patients who could not tolerate surgery due to poor general condition. (2) Patients with refractive turbid media and poor fixation. (3) Patients who received intrabulbar injection or other ophthalmic surgery. (4) Patients with other diseases causing macular edema. (5) Eyes with low quality OCTA images (image quality index <60) or media opacities, such as vitreous hemorrhage.

### Fundus Microvasculature Imaging by OCTA

The AngioVue system (Optovue RTVue XR100 Avanti; Optovue, Inc, Fremont, CA, USA) software (V.2017.1.0.155, Optovue, Fremont, CA, USA) was used for analyzing the OCTA images which were obtained before and after anti-VEGF treatment. The OCTA images were obtained at 7–11 am in the morning. The central fovea can be observed in each 6 × 6 mm image. In addition to the retina and choroid, the device software automatically depicts the structures of the SCP from DCP. The AngioVue software detects the SCP, consisting of a layer extending from 6 mm below the internal limiting membrane (ILM) to 15 μm below the inner plexiform layer (IPL), while the DCP extends from 15 to 70 μm below the IPL. Then, based on the collected images, VD in the SCP and DCP were automatically calculated as the percentage area occupied by blood vessels within 6 × 6 mm area, and the FAZ in the SCP and DCP were automatically calculated using the non-flow function in the software. If the segmentation lines were not correctly aligned according to the parameters defined above, we corrected them by using the manually correction of segmentation error. One expert grader evaluated the segmentation lines of the SCP and DCP (ILM, IPL, outer plexiform layer (OPL), and retinal pigment epithelium) in the registered horizontal OCT B-scan (18, 19). The “Edit Bnd/Propagation” option of the Optovue RTVue XR100 Avanti software (V.2017.1.0.155) was used for manually correction on the segmentation to the registered OCT B-scan and propagates the correction to the adjacent B-scans (18, 19). The segmentation correction was started from inner layers (ILM to OPL) on a single B-scan in the central fovea, and propagation function was automatically applied to correct the entire image for other B-scans. In addition, the measuring and comparing of the FAZ of each patient before and after IVC by automatic after manual correction measurement of OCTA (18, 19).

### Conbercept Treatment

Intravitreal injection of conbercept: levofloxacin eye drops were applied to prevent infection. Moreover, we recommend that patients use it 1 day before operation and 1 week after injection, and used it 4 times a day. After the conjunctival sac was flushed under surface anesthesia, a 30 GA syringe was used to inject 0.05 ml (0.5 mg) of conbercept into the vitreous. All patients were examined in the Shandong Eye hospital and received the treatment regime of intravitreal injection of conbercept. Patients initially received intravitreal conbercept injection for 3 months and 3 times a month and followed pro-re nata (PRN) injections treatment. If the patient showed a recurrence of macular edema and the central retinal thickness (CRT) exceeds 300 μm, the patient should be re-treated with conbercept in the 4th, 5th, and 6th month.

### Statistical Analyses

Our study was a randomized, single-masked analyze. The patient data included complete medical history and ophthalmic history: CRT, and retinal microvessels (mainly including VD and FAZ) were evaluated by OCTA before conbercept treatment and 1, 3, and 6 months after the conbercept treatment (one treatment per month, three consecutive times). The BCVA were evaluated before conbercept treatment and after the conbercept treatment for 6 months.

The SPSS (version 11.0) was used to calculate the mean and SD (mean ± SD) and *p* for all statistical data (^*^p < 0.05 and ^**^p < 0.01). The gender differences of all subjects were analyzed by *x*^2^ test. In addition, the age changes of all subjects were analyzed by *t*-test. When standard normal distribution was satisfied, the paired *t*-test was used to compare the pre-treatment and post-treatment after the conbercept treatment for 1, 3 months, or 6 months. But when the standard normal distribution was not satisfied, the Wilcoxon signed rank test was used. For analyzing the differences between groups (before 1st, 3rd, and 6th month), if normally distributed, we used ANOVA by ranks test. If it does not fit the normal distribution, we used Friedman's test.

## Results

In this prospective study, the information of 30 CI-DME with PDR patients and 30 NCI-DME with PDR patients were collected. However, a total of 17 patients were excluded, including 1 DME patient and 16 other patients with vitreous hemorrhage, macular fibrovascular hyperplasia, or media turbidity that may affect the image quality. Finally, 43 patients (27 men and 16 women) were included in this study. There were 29 cases (56 eyes) in CI-DME with PDR, including 37 eyes in 19 men and 19 eyes in 10 women, aged from 52 to 63 years (mean ± SD: 57.8 ± 3). There were 14 cases (26 eyes) in the NCI-DME with PDR, such as 16 eyes in 8 men and 10 eyes in 6 women, aged from 50 to 65 years (mean ± SD: 58.2 ± 5.5). The age and gender were not different between the patients with CI-DME and NCI-DME (Table 1). The patients with CI-DME had worse vision than NCI-DME (BCVA: 0.28 ± 0.08 vs. 0.38 ± 0.07, *p* < 0.05). The CRT in CI-DME group was thicker (426.46 ± 82.92 vs. 334.62 ± 12.23 μm, *p* < 0.05). The FAZ of NCI-DME was larger (0.31 ± 0.06 vs. 0.40 ± 0.32 mm^2^, *p* < 0.05).

**Table 1:** Baseline characteristics of participants.

**Variables**	**NCI-DME**	**CI-DME**	***p*** **value**
Number of patients	14 (26 eyes)	30 (56 eyes)	–
Male	8 (16 eyes)	19 (37 eyes)	–
Female	6 (10 eyes)	10 (19 eyes)	–
Age (mean ± SD), years	58.2 ± 5.5 (50–65)	57.8 ± 3 (52–63)	0.239
BCVA (mean ± SD)	0.38 ± 0.07	0.28 ± 0.08	<0.05
CRT (mean ± SD), μm	334.62 ± 12.23	426.46 ± 82.92	<0.05
FAZ (mean ± SD), mm^2^	0.40 ± 0.32	0.31 ± 0.06	<0.05

*BCVA, best corrected visual acuity; CRT, central retinal thickness; FAZ, foveolar avascular zone. p < 0.05, there is statistical difference*.

Conbercept was applied for intravitreal injection. The average injection times of patients with CI-DME and NCI-DME are 4.23 and 3.3, respectively (the injection times of all patients are between 3 and 5, *p* < 0.001). At the same time, we did not observe any serious complications during or after intravitreal therapy. Moreover, 6 months after intravitreal injections of conbercept, BCVA was increased in CI-DME group (0.28 ± 0.08 vs. 0.45 ± 0.12, *p* < 0.001), indicating that vision improved with conbercept treatment in the CI-DME group. However, conbercept treatment did not improve the vision in NCI-DME group (0.38 ± 0.07 vs. 0.42 ± 0.12, *p* = 0.058). After intravitreal injection of conbercept for 6 months, the CRT in the CI-DME and NCI-DME group were significantly lower compared with that in patients before the treatment (CI-DME: 426.46 ± 82.92 vs. 334.39 ± 96.55, *p* < 0.001; NCI-DME: 334.62 ± 12.23 vs. 295.58 ± 19.30, *p* < 0.001). After intravitreal injection of conbercept, there were no statistical differences in the FAZ. However, the reduction of FAZ in CI-DME was higher than that in patients with NCI-DME (0.12 vs. 0.07). Our results showed that the conbercept treatment improved the vision of CI-DME, and decreased the CRT in CI-DME. Therefore, conbercept is effective in the patients with PDR.

**Figure 1:**
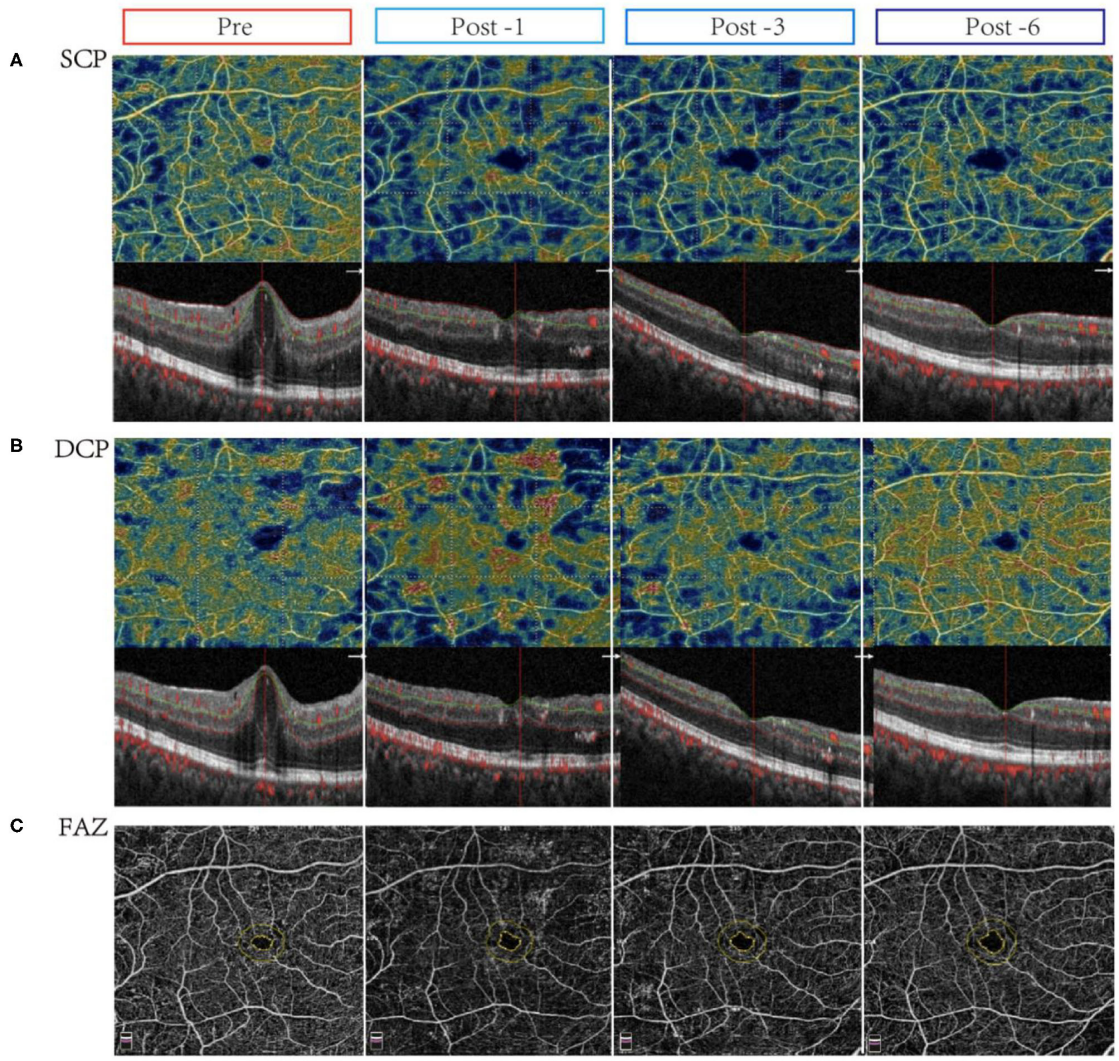
Changes in vessel density and flow area in eyes with center-involved diabetic macular edema (CI-DME) before and after intravitreal conbercept injection. Pre: before treatment; Post -1: 1 month after treatment; Post -3: 3 months after treatment; Post -6: 6 months after treatment. **(A)** The vessel density in the superficial capillary plexus (SCP) in patients with CI-DME before and after treatment for 1, 3, and 6 months: after treatment, the microvascular density in the inferior area of SCP improved. **(B)** The vessel density in the deep capillary plexus (DCP) in patients with CI-DME before and after treatment for 1, 3, and 6 months: there were no significant changes in different areas of DCP before and after treatment. **(C)** The foveolar avascular zone (FAZ) in patients with CI-DME before and after conbercept intravitreal injection: there was no significant difference before and after treatment.

Moreover, retinal microvascular changes after conbercept treatment were evaluated by OCTA. In CI-DME group, after treatment for 1 month, the VD of central fovea area in SCP had a slight reduction (*p* = 0.033). After 6 months of treatment, the VD in the inferior area of SCP was significantly increased (*p* = 0.002). There were significant differences in the VD of central fovea area in DCP between pre- and post-treatment (*p* < 0.001). However, there was no significant difference for VD in superior, inferior, nasal, tempo areas of DCP before and after treatment, even when the treatment was continued for 6 months (Table 2). Figures 1A–C shows the representative OCTA images of retinal VD and FAZ with CI-DME (pre-treatment vs. post-treatment).

**Table 2:** The change vessel density of superficial retinal capillary plexus and deep retinal capillary plexus_after conbercept treated CI-DME for 1, 3, and 6 months, compared to pre-treatment.

**CI-DME**		**Pre-treatment** **(*n* = 56)**	**Post-treatment (1)** **(*n* = 56)**	**Post-treatment (3)** **(*n* = 56)**	**Post-treatment (6)** **(*n* = 56)**	**Pre vs. Post -1** ***p* value**	**Pre vs. Post -3** ***p* value**	**Pre vs. Post -6** ***p* value**	**Rank test^#^**
SCP	-S	41.23 ± 6.79	43.64 ± 15.00	41.64 ± 5.21	42.27 ± 9.68	0.243	0.680	0.499	0.002^**^
	-I	41.90 ± 6.70	43.71 ± 15.12	41.06 ± 5.69	47.27 ± 4.89	0.092	0.393	0.002^**^	0.000^***^
	-N	42.41 ± 6.18	42.78 ± 16.23	41.67 ± 6.30	44.34 ± 5.08	0.863	0.476	0.179	0.023^*^
	-T	40.71 ± 6.31	42.87 ± 13.99	41.74 ± 5.33	41.24 ± 6.14	0.271	0.296	0.594	0.060
	-F	21.43 ± 6.83	18.55 ± 7.89	21.48 ± 4.13	21.82 ± 3.78	0.033*	0.624	0.790	0.003^**^
DCP	-S	42.16 ± 4.94	42.51 ± 4.47	46.13 ± 42.02	42.94 ± 5.08	0.564	0.481	0.374	0.474
	-I	42.03 ± 5.26	42.37 ± 4.63	48.81 ± 38.88	43.21 ± 6.08	0.601	0.196	0.251	0.000^***^
	-N	42.26 ± 4.87	42.54 ± 4.65	48.70 ± 41.09	43.21 ± 3.61	0.647	0.258	0.256	0.578
	-T	41.60 ± 5.32	41.31 ± 4.86	49.22 ± 37.94	44.36 ± 37.97	0.714	0.153	0.601	0.000^***^
	-F	28.99 ± 6.97	19.58 ± 9.11	22.96 ± 3.77	23.00 ± 3.64	0.000^***^	0.000^***^	0.000^***^	0.000^***^
CRT	-F	426.46 ± 82.92	393.39 ± 68.13	392.58 ± 62.94	334.39 ± 96.55	0.000^***^	0.000^***^	0.000^***^	0.000^***^

*We used the paired t-test and Wilcoxon signed rank test test to analyze the effect of Conbercept intravitreal injection acted on CI-DME before treatment and 1, 3 and 6 months after treatment. And rank test was used to analyze the differences between groups (*

# *, Friedman test). These were tested in SCP, superficial retinal capillary plexus; DCP, deep retinal capillary plexus; CRT, central retinal thickness; S, segmented from superior; I, inferior; N, nasal; T, tempo; F, fovea. Pre-treatment/Pre, before tretment; Post-treatment (1)/Post (1), one month after treatment; Post-treatment (3)/Post (3), three month after treatment; Post-treatment (6)/Post (6), six month after treatment*.

* *p < 0.05*,

** *p < 0.01*,

*** *p < 0.001*.

**Figure 2:**
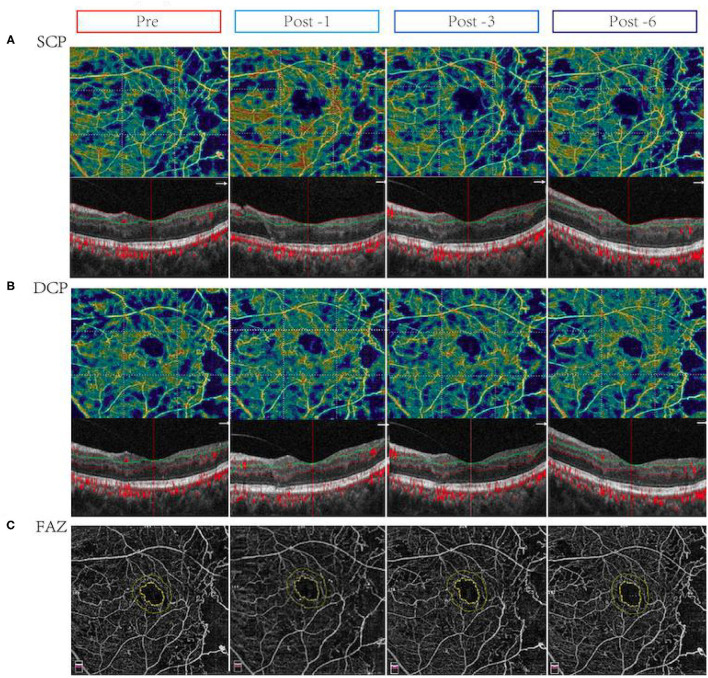
Changes in vessel density (VD) and flow area in eyes with non-center involved DME (NCI-DME) before and after intravitreal conbercept injection. Pre: before treatment; Post -1, 1 month after treatment; Post -3, 3 months after treatment; Post -6, 6 months after treatment. **(A)** The vessel density in the superficial capillary plexus (SCP) before and after treatment for 1, 3, and 6 months with NCI-DME patients: after treatment, the microvascular density in the inferior area of SCP improved. **(B)** The vessel density in the deep capillary plexus (DCP) in patients with NCI-DME before and after treatment for 1, 3, and 6 months: after treatment, the microvascular density in the central fovea area of DCP improved. **(C)** The FAZ in patients with NCI-DME before and after conbercept intravitreal injection: there was no significant difference before and after treatment.

However, in NCI-DME group, the VD in inferior area of SCP was increased 6 months after conbercept treatment (Table 3). After treatment for 3 months, there were significant differences in the nasal, tempo, and central fovea areas of DCP. All parameters of the VD in the whole images and regions of DCP in NCI-DME group were significantly changes after 6 months conbercept treatment (Table 3). Representative OCTA images demonstrating the changes in VD and FAZ with NCI-DME group (pre-treatment vs. post-treatment) are shown in Figures 2A–C. Above all, the conbercept treatment affects the DCP of NCI-DME group.

**Table 3:** The change vessel density of superficial retinal capillary plexus and deep retinal capillary plexus after conbercept treated NCI-DME for 1, 3, and 6 months, compared to pre-treatment.

**NCI-DME**		**Pre-treatment** **(*n* = 26)**	**Post-treatment (1)** **(*n* = 26)**	**Post-treatment (3)** **(*n* = 26)**	**Post-treatment (6)** **(*n* = 26)**	**Pre vs. Post -1** ***p* value**	**Pre vs. Post -3** ***p* value**	**Pre vs. Post -6** ***p* value**	**Rank test^**#**^**
SCP	-S	43.69 ± 5.59	47.43 ± 18.64	43.43 ± 7.16	44.76 ± 5.42	0.567	0.852	0.128	0.089
	-I	42.23 ± 4.86	48.19 ± 17.17	44.53 ± 4.75	47.23 ± 4.89	0.014^*^	0.021^*^	0.005^**^	0.000^***^
	-N	43.76 ± 4.26	47.23 ± 15.87	42.94 ± 6.21	45.33 ± 4.95	0.422	0.970	0.193	0.403
	-T	42.69 ± 5.11	45.69 ± 15.62	43.91 ± 9.50	44.52 ± 4.47	0.193	0.429	0.435	0.009^**^
	-F	19.53 ± 6.49	19.35 ± 10.14	20.92 ± 5.24	21.23 ± 5.45	0.374	0.394	0.150	0.000^***^
DCP	-S	42.99 ± 2.94	43.30 ± 3.72	44.16 ± 5.62	40.25 ± 3.68	0.711	0.216	0.000^***^	0.000^***^
	-I	43.08 ± 3.46	43.15 ± 4.09	44.46 ± 3.86	40.23 ± 3.21	0.576	0.137	0.000^***^	0.000^***^
	-N	42.85 ± 2.57	43.41 ± 3.63	44.31 ± 4.28	40.15 ± 3.28	0.474	0.029^*^	0.000^***^	0.000^***^
	-T	43.09 ± 4.33	43.99 ± 5.56	44.61 ± 3.91	39.81 ± 3.74	0.162	0.013^*^	0.011^*^	0.000^***^
	-F	16.58 ± 7.61	15.91 ± 7.13	22.12 ± 3.75	21.88 ± 3.39	0.567	0.002^**^	0.000^***^	0.005^**^
CRT	-F	334.62 ± 12.23	329.15 ± 19.84	331.34 ± 20.16	295.58 ± 19.30	0.135	0.437	0.000^***^	0.000^***^

*We used the paired t-test and Wilcoxon signed rank test test to analyze the effect of Conbercept intravitreal injection acted on NCI-DME before treatment and 1, 3 and 6 months after treatment. And rank test was used to analyze the differences between groups (*

# *, Friedman test). These were tested in SCP, superficial retinal capillary plexus; DCP, deep retinal capillary plexus; CRT, central retinal thickness; S, segmented from superior; I, inferior; N, nasal; T, tempo; F, fovea. Pre-treatment/Pre, before tretment; Post-treatment (1)/Post -1, one month after treatment; Post-treatment (3)/Post -3, three month after treatment; Post-treatment (6)/Post -6, six month after treatment*.

* *p < 0.05*,

** *p < 0.01*,

*** *p < 0.001*.

## Discussion

Our results suggested that vision has significantly improved before and after intravitreal injection of conbercept in the CI-DME group. Some clinical trials have demonstrated that anti-VEGF therapy has been adopted as the first-line therapy in CI-DME (5–7, 27) and it is more effective in improving the vision and visual acuity in CI-DME compared with focal laser treatment. Anti-VEGF treatment blocked the formation of neovascularization and reduced retinal thickening (5, 26, 27). In addition, multiple intravitreal conbercept in patients with DME may improve macular VD. Our study further showed the effect of conbercept on the SCP and DCP of CI-DME and NCI-DME. Regardless of the central fovea involvement, the conbercept improved microvascular density in DME, which affected VD in retinal capillary plexus which may be the mechanism of the drug. The conbercept treatment improved the microvascular density in multiple regions of NCI-DME, which are more than that of CI-DME. Thus, our results showed that the intravitreal conbercept was effective on PDR and DME. Furthermore, the conbercept treatment can decrease the retinal thickness and reduce the area of macular edema.

As a new non-invasive and high-resolution fundus angiography technology, OCTA is extremely useful in the quantitative analysis of macular VD and segment detection of FAZ (2). In our study, the FAZ of patients with PDR (before and after intravitreal injection of conbercept treatment) was observed by OCTA. This technique is very effective in revealing vascular abnormalities, such as neovascularization on the surface of the retina and optic nerves, and OCTA has also been applied to the diagnosis, treatment, and follow-up of various fundus vascular diseases (22–24). However, it is not capable of visualizing leakage and completely avoiding stepwise segmentation error, which could be construed as limitations (18–21, 28).

In addition, in previous research, anti-VEGF drugs, such as bevacizumab, ranibizumab, aflibercept, and conbercept were effective for DME (14, 29). However, there has been controversy about whether the macular perfusion will get better or worse after anti-VEGF treatment (15, 22–24, 30). Zhao et al. considered that there were no significant changes of macular and papillary VD after either the panretinal photocoagulation or intravitreal conbercept treatment (23). However, with the progress of DR and the decrease of VD, both treatments may prevent the loss of macular and papillary VD in PDR. Although there are normal diurnal variations of macular thickness/perfusion (31, 32), some studies considered that anti-VEGF treatment may have a positive effect on macular perfusion status, such as the improvement of BCVA and the reduction of neovascularization (2, 22, 24). Conbercept treatment was proved to affect the superficial macular VD (15, 23). Our study proved that intravitreal conbercept treatment improved the vision and VD in patients with DME.

However, there are some limitations to our study which are as follows: (1) there were biases in the selection of patients. OCTA was used to observe the fundus, so the patients with poor vision and poor fixation were excluded. (2) Lacking the data on deep FAZ and ignoring diurnal variations in choroidal sublayer perfusion. (3) The number of cases was limited and the follow-up time was short. Therefore, in future studies, we need to increase the sample size, the number, and the follow-up time and prolong the treatment time to support our research.

In conclusion, OCTA is a non-invasive and promising emerging technique, which can be used to obtain quantitative data and more detailed information of the SCP or DCP vascular network in the eyes of patients with CI-DME and NCI-DME. We found that conbercept effectively relieved macular edema and improved vision.

## Data Availability Statement

The original contributions presented in the study are included in the article/Supplementary Material, further inquiries can be directed to the corresponding author/s.

## Ethics Statement

This Human Research was approved by the Institutional Ethics Committee of Shandong Eye Hospital (Approval No. 2019S001) on November 16, 2019. The patients/participants provided their written informed consent to participate in this study. Written informed consent was obtained from the individual(s) for the publication of any potentially identifiable images or data included in this article.

## Author Contributions

TiL and WLin designed and conducted clinical examinations. TiL, WLin, and MF wrote and revised the manuscript. QW and ZM collated the clinical data. WW, XX, and WLi analyzed and interpreted the data. XX, WW, JG, and ToL collated and drew the figures and tables. QZ gave guidance and put forward suggestions for revision. All authors contributed to the revision steps and approved the final version of the manuscript for submission.

## Funding

This study was supported by a grant from the Natural Key Research and Development Project (2016YFC1305500) and Bethune Langmu Young Scholars Research Fund (BJ-LM2021007J).

## Conflict of Interest

The authors declare that the research was conducted in the absence of any commercial or financial relationships that could be construed as a potential conflict of interest.

## Publisher's Note

All claims expressed in this article are solely those of the authors and do not necessarily represent those of their affiliated organizations, or those of the publisher, the editors and the reviewers. Any product that may be evaluated in this article, or claim that may be made by its manufacturer, is not guaranteed or endorsed by the publisher.
